# Inflammation and Cognition in Children and Adolescents: A Call for Action

**DOI:** 10.3389/fped.2020.00583

**Published:** 2020-09-09

**Authors:** Mireia Adelantado-Renau, Maria Reyes Beltran-Valls, Diego Moliner-Urdiales

**Affiliations:** LIFE Research Group, University Jaume I, Castellon, Spain

**Keywords:** inflammatory factors, cognitive function, health, childhood, adolescence

## Introduction

Inflammation is a natural response to injury or infection, which promotes tissue survival, remodeling, and repair, as well as adaptation to stress and restoration of the homeostatic state (1). In the acute phase of an inflammatory response, which could persist for a few days, inflammatory factors such as cytokines/chemokines, immune-related effectors, acute phase proteins, and reactive oxygen and nitrogen species are released, thereby triggering coordinated biological events (1). However, a prolonged inflammatory response, also known as chronic low-grade inflammation (1), may contribute to the pathogenesis of several cardiovascular and metabolic diseases (e.g., atherosclerosis, diabetes, and cancer) (2, 3) as well as to the development of neuropsychiatric disorders and cognitive dysfunctions (4).

Cognition involves a set of mental processes that shape perception, memory, intellect, and action, including executive functions (e.g., cognitive inhibition, cognitive flexibility, and working memory), and other cognitive domains (e.g., verbal fluency and comprehension) (5). Although it has been suggested that the immune system might modulate brain functioning, how inflammatory factors could influence cognition is poorly understood. Here, we provide context and three open questions that need to be answered to move the field forward.

### Is Low-Grade Systemic Inflammation Associated With Cognition in Children and Adolescents?

The current understanding of the association between systemic inflammation, which takes place outside the central nervous system, and cognition has been mainly focused on the two endpoints of the human life span as well as on psychiatric patients. In extremely preterm infants, incremented levels of inflammatory proteins (e.g., C-reactive protein) have been related to impaired cognitive function later in life (6, 7). Interestingly, prenatal inflammation, measured by interleukin-6, has also been shown to have an impact on postnatal cognitive outcomes (8). In older adults, there is a link between incremented levels of proinflammatory cytokines (e.g., interleukin-6, interleukin-1β, and tumor necrosis factor-α) and neurodegenerative disorders, such as Alzheimer's disease and other dementias (9). In psychiatric patients, a growing body of evidence has shown that high levels of proinflammatory cytokines and C-reactive protein are associated with altered cognition in individuals with neuropsychiatric disorders, such as schizophrenia, bipolar disorder, and major depressive disorders (10). Additionally, prior literature has pointed out that a state of chronic low-grade systemic inflammation could also negatively influence cognition in healthy midlife adults (11).

In contrast to this prior evidence, the association between inflammation and cognition, both assessed during childhood and adolescence (4–18 years), is poorly understood since only six studies have been conducted in this age group, showing inconclusive results (12–17). Concurring with research in other age range populations, four studies have shown that elevated levels of circulating inflammatory factors in children and adolescents are associated with impaired cognitive processes (12–14, 16). Conversely, two studies reported null associations between these parameters (15, 17). This previous research focused on only some immune-related effectors, cytokines, and C-reactive protein. With regard to cognition, studies have mainly investigated memory (12, 15–17) and a global measure of executive function (12, 14–16), with only a few studies examining other cognitive domains, such as cognitive inhibition (16, 17), cognitive flexibility (17), or attention (14). The paucity of studies and the methodological differences among them (e.g., selected signaling proteins and cognitive-related outcomes, and study design) prevent drawing firm conclusions and make necessary additional research on this interesting and promising area.

During childhood and adolescence, brain regions are continuously developing (18), which shapes the cognitive domains. Indeed, these periods of life provide a developmental window with a great opportunity to experience structural and functional organization of the brain influenced by exogenous and endogenous factors (19), for example, inflammation. Therefore, future studies analyzing the impact of a wide range of inflammatory factors on the whole range of cognitive outcomes in children and adolescents are needed to advance our understanding of this Research Topic.

### How Could Inflammation Influence Complex Higher-Order Neurological Functions?

Inflammatory factors could influence cognition through two different routes. Firstly, systemic inflammatory proteins can enter the brain by crossing the blood–brain barrier via active transport mechanisms or via vagal nerve stimulation (20). Secondly, within the brain, inflammatory factors can be expressed by astroglia, microglia, neurons, and endothelial cells as a consequence of brain injury, trauma, or intracerebral infections (21). In animal models, both systemic and central inflammation may alter cognition through its effects on synaptic plasticity, neurotransmission, and dendritic branching (22). In humans, clinical research has suggested that systemic inflammatory agents may play a substantial role in neurodevelopmental processes, such as synaptic plasticity, neurogenesis, and neuromodulation (21). Therefore, we speculate that inflammation alters these brain functions, which ultimately leads to alterations in cognitive functioning. The impact of inflammation on cognition could be indirectly related to several cardiometabolic disorders (e.g., obesity, type 2 diabetes, and hypertension), since the pathogenesis of these disorders is closely linked to chronic low-grade systemic inflammation (2, 3). However, the exact molecular and cellular pathways by which inflammatory factors may influence cognition remain to be elucidated. It is likely that inflammatory agents exert unique effects in different cortical regions and may have specific consequences on the brain regions depending on the state of brain development (23). Previous studies in humans, which included brain imaging data, have suggested that peripheral proinflammatory cytokines, such as interleukin-6, interleukin-1β, and tumor necrosis factor-α, are associated with changes in functional connectivity as well as in cortical thickness and surface area in specific regions of the brain (23, 24). In this sense, further longitudinal studies including brain imaging data, which could detect brain function and structural adaptations, may contribute to a better understanding of the effect of inflammation on cognition over a period of time. In addition, further studies could also analyze brain functions as mediator mechanisms involved in the association between inflammation and cognition.

### How Could Inflammation Be Modulated to Improve Cognition?

Therapeutic or prophylactic immunomodulation is a strategy that activates the non-specific immune system to counteract inflammation (25). Alternatively, prior evidence has suggested that lifestyle behaviors, such as diet, physical activity, or sleep, could act as preventive strategies influencing the inflammatory levels, since associations between these behaviors and inflammation have been reported in children and adolescents (26, 27). For instance, physical activity affects inflammatory mediators by the reduction of visceral fat mass, improvement of endothelial function and insulin sensitivity, and elevation of anti-inflammatory cytokines levels and other muscle contraction-derived proteins (28).

Considering that childhood and adolescence are crucial periods of life in terms of establishing healthy lifestyle behaviors and acquisition of cognitive skills, the investigation of the influence of these modifiable behaviors on inflammation and cognitive functioning is of paramount importance. Therefore, from a public health perspective, further prospective cohort studies and non-pharmacological randomized lifestyle interventions focusing on inflammation as a primary outcome could help to predict the direction of causality and to understand its role in cognition in youths. In addition, since the mechanisms underlying these associations have not been clearly identified, we believe that inflammatory-mediated pathways in relation to cognition should be considered as a new and exciting Research Topic.

## Conclusion

Since high levels of inflammatory factors during childhood and adolescence, considered important periods of life in terms of brain development, seem to track into adulthood (29), the promotion of health is particularly needed. Thus, further research is warranted to not only facilitate our understanding of neural-immune communication pathways and cytokine-mediated pathophysiological processes but also to design efficient non-pharmacological lifestyle intervention programs to tackle inflammation-related diseases. These lifestyle interventions may be cost-effective, overcoming the benefits of pharmacological approaches (e.g., anti-inflammatory drugs, immunomodulators, or nutraceuticals), since they may exert multi-systemic positive effects on health with little contraindications and at a lower cost. Moreover, lifestyle interventions could be used not only as treatment strategies but also as primary prevention strategies. Future studies should consider participants' maturational status to account for individual developmental features. In addition, studies should include sample sizes large enough to provide significant statistical power as well as guarantee participants' compliance. Specifically, lifestyle interventions focused on physical activity should precisely define its dose in terms of intensity, type, and duration in order to facilitate comparisons among studies. With regard to diet, interventions should focus not only on dietary patterns but also on the intake of specific foods, nutrients, and non-nutrient food components. Lastly, sleep interventions aimed at modulating inflammation through the improvement of sleep patterns should also be considered.

Based on previous scientific literature, low-grade systemic inflammatory proteins could be identified as critical factors for cognitive functioning during youth. We believe that the scientific community should be aware of the gap highlighted in this manuscript in order to take action and expand the knowledge on inflammation and cognition in the young population.

## Author Contributions

MA-R was involved in the manuscript preparation and drafting of the initial manuscript. MRB-V and DM-U were involved in the manuscript preparation and critical revision. All authors contributed to the article and approved the submitted version.

## Conflict of Interest

The authors declare that the research was conducted in the absence of any commercial or financial relationships that could be construed as a potential conflict of interest.
